# A Quack Remedy for Hydrophobia

**Published:** 1855-09

**Authors:** 


### A QUACK REMEDY FOR HYDROPHOBIA.

For more than forty years, a family by the name of Marchand, residing in western Pennsylvania, has had great notoriety for making and vending a nostrum for the prevention and cure of hydrophobia. Quite recently I had an opportunity of examining this nostrum, and send you the result.

The potion consists of three boluses, and in each bolus is a pellet of paper closely rolled. On unrolling the pellet carefully, I was enabled to read the following words, written in a fair hand: "Margarat, Feragat, Magulat." Of course the efficacy of the bolus resides in the magical words.

A fatal case of hydrophobia occurred last month in Allegheny the friends had procured the Marchand nostrum, and I was thus enabled to see the bolus for the first time.

JOS. P. GAZZAM, M. D.

Pittsburgh, October, 1846.—*Med. News and Library.*

We had occasion in a former number of this work to refer to the numerous quack remedies used for the cure of hydrophobia. As far back as our recollection goes we remember to have heard of the boasted powers of this family in which there were several brothers who were physicians, all of whom claimed this power, but was kept concealed by them. This potent remedy we remember too was used, to the exclusion of all else, in the case of a Mr. M. Cammant, who was bitten by a rabid wolf, of which he died in fearful demonstrations of agony and suffering, "Margarat, Feragat, Magulat" to the contrary notwithstanding. One of these Marchands lived in Uniontown, Pennsylvania, another on the Monongahela below Brownsville, and we think there was another of these disciples of Margarat, & Co. somewhere dispensing his pellets. These men were uroscopists too and could do marvellous things even almost to raising the dead. They have been described to us as highly competent to carry on a successful system of chicanery, being smooth, sententious, retired, and wonderfully knowing. With this magical nostrum alone they doubtless amassed a fortune and with it too they as surely cured numbers who were bitten by *dogs not a whit rabid.* Uniontown was found to be a congenial spot for the success of empirics. A fellow by the name of Braddee who was strongly suspected of being connected with the great land pirate Merril, figured there conspicuously for a time, during which he amassed a large estate. In every art of cunning he proved himself an adept, and it was astonishing the hold, which by his duplicity and deception, he had acquired upon the confidence of persons who made claims to intelligence and respectability. His great element of success lay in his vain boasting and the capacity of looking grave and profoundly sincere in his boasted powers.—Although amassing money he was not content with this kind of swindling, but it was believed he joined a gang of counterfeiters and prosecuted that scheme of money making which was little less honest than his other calling and pursuit. His pirate habits and his proclivities for plunder became too strong for longer restraint, and incendiarism and mail robbing were perpetrated so frequently, that finally he was arrested, convicted of the latter, and sent to the State's prison, where he terminated his career of infamy and crime.

But we ever felt a curiosity to know the ingredients of this celebrated Marchand nostrum and feel that we owe a debt of gratitude to Dr. Gazzam for his discovery and revelation.

